# Psychosocial development in survivors of childhood differentiated thyroid carcinoma: a cross-sectional study

**DOI:** 10.1530/EJE-17-0741

**Published:** 2017-12-18

**Authors:** Marloes Nies, Bernadette L Dekker, Esther Sulkers, Gea A Huizinga, Mariëlle S Klein Hesselink, Heleen Maurice-Stam, Martha A Grootenhuis, Adrienne H Brouwers, Johannes G M Burgerhof, Eveline W C M van Dam, Bas Havekes, Marry M van den Heuvel-Eibrink, Eleonora P M Corssmit, Leontien C M Kremer, Romana T Netea-Maier, Heleen J H van der Pal, Robin P Peeters, John T M Plukker, Cécile M Ronckers, Hanneke M van Santen, Anouk N A van der Horst-Schrivers, Wim J E Tissing, Gianni Bocca, Thera P Links

**Affiliations:** 1 Division of EndocrinologyDepartment of Internal Medicine; 2 Department of Wenkebach InstituteSchool of Nursing and Health and Beatrix Children’s Hospital; 3 Department of Pediatric OncologyUniversity of Groningen, Beatrix Children's Hospital, University Medical Center Groningen, Groningen, the Netherlands; 4 Psychosocial DepartmentAcademic Medical Center, Amsterdam, the Netherlands; 5 Princess Máxima Center for Pediatric OncologyUtrecht, the Netherlands; 6 Department of Nuclear Medicine and Molecular Imaging; 7 Department of EpidemiologyUniversity of Groningen, University Medical Center Groningen, Groningen, the Netherlands; 8 Division of Endocrinology, Department of Internal MedicineVU University Medical Center, Amsterdam, the Netherlands; 9 Division of Endocrinology, Department of Internal MedicineMaastricht University Medical Center, Maastricht, the Netherlands; 10 Department of Pediatric OncologyRotterdam, the Netherlands; 11 Division of Endocrinology, Department of Internal MedicineLeiden University Medical Center, Leiden, the Netherlands; 12 Department of Pediatric OncologyAcademic Medical Center, Amsterdam, the Netherlands; 13 Division of Endocrinology, Department of Internal MedicineRadboud University Medical Center, Nijmegen, the Netherlands; 14 Department of Medical Oncology and Emma Children’s Hospital/Academic Medical CenterAmsterdam, the Netherlands; 15 Department of Internal Medicine and Rotterdam Thyroid Center Erasmus Medical CenterRotterdam, the Netherlands; 16 Department of Surgical OncologyUniversity of Groningen, University Medical Center Groningen, Groningen, the Netherlands; 17 Department of PediatricsUniversity Medical Center Utrecht, Wilhelmina Children’s Hospital, Utrecht, the Netherlands; 18 Department of Pediatric EndocrinologyUniversity of Groningen, Beatrix Children's Hospital, University Medical Center Groningen, Groningen, the Netherlands

## Abstract

**Objective:**

The impact of childhood differentiated thyroid carcinoma (DTC) on psychosocial development has not yet been studied. The aim of this study was to evaluate the achievement of psychosocial developmental milestones in long-term survivors of childhood DTC.

**Design and methods:**

Survivors of childhood DTC diagnosed between 1970 and 2013 were included. Reasons for exclusion were age <18 or >35 years at follow-up, a follow-up period <5 years or diagnosis with DTC as a second malignant neoplasm. Survivors gathered peer controls of similar age and sex (*n* = 30). A comparison group non-affected with cancer (*n* = 508) and other childhood cancer survivors (CCS) were also used to compare psychosocial development. To assess the achievement of psychosocial milestones (social, autonomy and psychosexual development), the course of life questionnaire (CoLQ) was used.

**Results:**

We included 39 survivors of childhood DTC (response rate 83.0%, mean age at diagnosis 15.6 years, and mean age at evaluation 26.1 years). CoLQ scores did not significantly differ between survivors of childhood DTC and the two non-affected groups. CoLQ scores of childhood DTC survivors were compared to scores of other CCS diagnosed at similar ages (*n* = 76). DTC survivors scored significantly higher on social development than other CCS, but scores were similar on autonomy and psychosexual developmental scales.

**Conclusions:**

Survivors of childhood DTC showed similar development on social, autonomy, and psychosexual domains compared to non-affected individuals. Social development was slightly more favorable in DTC survivors than in other CCS, but was similar on autonomy and psychosexual domains.

## Background

Childhood diagnosis of thyroid cancer is rare. Age-adjusted incidence rates of pediatric thyroid cancer are reported between 0.6 and 1.2 per 100 000 per year, but rates are increasing (1, 2, 3). Differentiated thyroid carcinoma (DTC) is the most frequently diagnosed histological subtype of this disease, accounting for over 90% of the thyroid cancers in children (1). The incidence of childhood DTC peaks during puberty and mainly affects girls (4, 5). The effect of DTC on the psychosocial development of children diagnosed with this disease has not been studied.

Childhood cancer arises in a period where several psychosocial developmental milestones are reached (6), also referred to as ‘the course of life’ (7). The psychosocial development from childhood toward adulthood involves increasing independence, growth of more symmetrical parent–adolescent relationships, maturation of personality and identity, and encountering and maintaining (first) psychosexual and social contacts (8). The diagnosis and treatment of childhood cancer may impair the psychosocial development as some childhood cancer survivors (CCS) have been shown to be hindered in their development. For example, CCS had fewer friends and tended to be older when they experienced their first sexual intimacy. However, data are inconclusive (7, 9, 10, 11, 12). In survivors of various types of childhood cancer the biological behavior of the cancer and the impact of treatment interfere differently in the course of life: survivors of brain tumors seem most severely affected (9, 10, 11, 13).

Unfortunately, despite the excellent cure rates, most DTC survivors remain patients for the rest of their lives. Treatment of DTC consists of a thyroidectomy, and in case of lymph node metastases, it is extended with a lymph node dissection in the neck. In the Netherlands, about a third of the survivors of childhood DTC experience surgical complications: the most common complications are hypoparathyroidism and recurrent laryngeal nerve damage (5). Surgery is followed by one or more administrations of radioactive iodine (131-I). During the 131-I administrations, patients are withdrawn from thyroid hormone for several weeks; this causes deep hypothyroidism, involving changes in metabolism (14). Initial treatment usually takes from one to several years. Subsequently, lifelong thyroid hormone therapy is initiated. Especially for high-risk patients, relatively high levels of thyroid hormone are prescribed to induce subclinical hyperthyroidism, which decreases the risk of cancer recurrence (15). However, large fluctuations in thyroid hormone levels are known to have a great impact on well-being, behavior, and learning ability (16, 17). Childhood DTC has an excellent survival, with 5-year and 10-year survival rates >95% (4, 5). Furthermore, specific aspects of DTC survivorship include lifelong use of hormone medication and blood tests as well as the possibility of cancer recurrences even after decades of disease-free survival (18).

An explorative study was set up, ultimately to evaluate whether interventions for these survivors are necessary. Our aims were (I) to compare the developmental milestones reached by adult survivors of childhood DTC and by individuals non-affected with cancer; (II) to compare psychosocial development in adult survivors of childhood DTC and in survivors of other types of childhood cancer and (III) to assess whether the psychosocial development of survivors of childhood DTC is associated with medical characteristics.

## Subjects and methods

This cross-sectional study was performed in the context of a Dutch nationwide study examining medical characteristics and long-term treatment effects (including a paper questionnaire) of childhood DTC from 2012 to 2014 (5). The Institutional Review Board of the University Medical Center Groningen approved the study on behalf of all participating institutions (ABR NL40572.042.12, file number 2012/183). This study was registered in the Netherlands Trial Registry (trial registration number 3448).

### Participants

#### DTC survivors

For the nationwide study, all patients diagnosed with DTC at age ≤18 years between 1970 and 2013 treated in the Netherlands were included (5). Exclusion criteria were, as previously reported: follow-up <5 years after diagnosis, attained age <18 years, diagnosis of DTC as a second malignant neoplasm, lack of understanding of the Dutch language, and 131-I administration within three months before evaluation (19). For the current study, survivors aged >35 years during evaluation were also excluded because the questionnaire is validated only up to the age of 35 years (7).

### Groups used for comparison

#### Comparison to individuals unaffected by cancer

DTC survivors were compared to a group of persons unaffected by cancer from the survivors’ environment (peer controls) and compared to an unaffected group reflecting the general population (comparison group).

*Peer controls*: DTC survivors were asked to find one or two peers of the same sex and age (range plus or minus 5 years from the survivor’s age at evaluation, minimum age 18 years). Controls who had a medical history of malignancy were excluded. Although we preferred peers (e.g. friends, colleagues), we allowed inclusion of family members, provided they met the in/exclusion criteria (19). For the current substudy, peer controls aged >35 years were also excluded.

All DTC survivors and peer controls gave written informed consent before participating in the study.

*Comparison group*: A comparison group, gathered by general practitioners in a previous study, was used (7). For this comparison, group persons aged 18–30 years, with no history of cancer and with the ability to understand Dutch questionnaires were included. This group consisted of 508 persons (consisting of 53% females and 47% males).

#### Comparison to survivors of other childhood cancers

*Survivors of other childhood cancers*: For this comparison, we used data of other CCS, gathered in a study by Stam and coworkers (7). These survivors were diagnosed with cancer at age <18 years, were at least 5 years in follow-up, aged 17–30 years during evaluation, and were able to understand Dutch questionnaires. The different cancer diagnoses were grouped as leukemia/lymphoma (e.g. acute lymphoblastic leukemia, (non-) Hodgkin lymphoma), solid tumors (e.g. rhabdomyosarcoma, osteosarcoma) and brain tumors (7). Three hundred and fifty-three CCS were eligible for comparison. Survivors of thyroid cancer (*n* = 3) were excluded from analyses since we only wanted to compare DTC survivors to other types of childhood cancer. Subsequently, 350 CCS of other types of cancer (hereafter referred to as CCS) were available for comparison.

### Measures

#### Psychosocial development

Psychosocial development was assessed using the course of life questionnaire (CoLQ) (7), which measures achievement of developmental milestones on five domains: (1) social development, (2) autonomy development, (3) psychosexual development, (4) antisocial behavior, and (5) substance use and gambling. To restrict the length of the questionnaire, for the current study only domains 1–3 were evaluated. Possible scores for each item were scored 1 (milestone not (yet) achieved) or 2 (milestone achieved). Scores were added-up to form 3 scales: social development (12 items, range: 12–24), autonomy development (6 items, range: 6–12) and psychosexual development (4 items, range: 4–8). Higher scores indicate earlier achievement or achievement of more developmental milestones. The validity of the course of life scales is good. The internal consistency of the scales is satisfactory, except the autonomy scale, probably because the items refer to diverging aspects of autonomy (7). The Cronbach’s alphas in the populations under study were moderate to good: (1) social development (range: 12–24): DTC survivors 0.70, peer controls 0.76, comparison group 0.71, CCS 0.75; (2) autonomy development (range: 6–12): DTC survivors 0.52, peer controls 0.49, comparison group 0.49, CCS 0.43; (3) psychosexual development (range: 4–8): DTC survivors 0.91, peer controls 0.75, comparison group 0.71, CCS 0.74.

#### Sociodemographic and medical data

Sociodemographics (marital status, educational level (low, medium and high) and employment status) and data regarding disease, treatment, and follow-up characteristics were retrieved from medical records and a self-administered questionnaire.

### Statistical methods

Data are presented as median (interquartile range) unless otherwise specified. Characteristics and scores on the CoLQ (on scale level and item level) of survivors of childhood DTC were compared separately with the characteristics or scores of the peer controls, the comparison group and the CCS (total, and divided by type of diagnosis). Associations between medical characteristics and scores on developmental domains were evaluated.

*χ* ^2^ tests or Fisher’s exact tests (if >20% of the cells had an expected count of <5) were used for categorical variables. Mann–Whitney *U* and Kruskal–Wallis tests were performed for non-normally distributed continuous or ordinal variables. Spearman’s rank correlation coefficient was used to correlate two non-normally distributed continuous and/or ordinal variables. The tests performed are described in the table legends. Missing or unknown values were excluded from statistical testing (pairwise deletion). To compensate for multiple testing, we considered differences to be statistically significant at *P* < 0.01. All tests were two-sided. IBM SPSS Statistics for Windows, version 23 (IBM) was used for statistical analyses.

## Results

### I) Comparison to individuals unaffected with cancer

#### Sample characteristics

One hundred and five survivors were included in the nationwide study (of 169 eligible subjects, response rate 62.1%) (5). Of the 47 survivors eligible for the current study, 39 (83.0%) participated (Supplementary Fig. 1, see section on supplementary data given at the end of this article). The survivors gathered 59 peer controls, 30 of whom participated: 26 peer controls were excluded because of an age >35 years and 3 peer controls were excluded because they did not meet matching criteria.

Demographic characteristics appear in Table 1. Median age of the DTC survivors at evaluation was 26.2 years (range 18.8–35.7), compared to a median age of 25.8 years (range 19.4–34.4, *P* = 0.628) of peer controls and a median age of 23.8 years of the comparison group (range 18.0–31.0, *P* = 0.012). The group of DTC survivors had significantly more females than the comparison group (87% vs 53%,* P* < 0.001). DTC survivors reported significantly more frequently higher levels of education than the comparison group (*P* = 0.002). Other characteristics did not significantly differ between DTC survivors and the other two groups.

**Table 1:** Characteristics of survivors of childhood DTC vs individuals non-affected with cancer.

	DTC survivors	Peer controls	*P* value	Comparison group	*P* value
*n*	39	30		508	
Age at evaluation (years)	26.2 (18.8–35.7)	25.8 (19.4–34.4)	0.628^†^	23.8 (18.0–31.0)	0.012^†^
Sex, *n* (%)			0.690^‡^		<0.001*^§^
Female	34 (87)	28 (93)		269 (53)	
Male	5 (13)	2 (7)		239 (47)	
Employment, *n* (%)			0.576^‡^		1.000^‡^
Employed and/or student	38 (97)	28 (93)		486 (96)	
Not employed and no student	1 (3)	2 (7)		21 (4)	
Missing	0 (0)	0 (0)		1 (0)	
Completed education, *n* (%)			0.035^‡^		0.002*^§^
Low level	7 (18)	0 (0)		143 (28)	
Medium level	15 (39)	12 (40)		246 (48)	
High level	17 (44)	18 (60)		97 (19)	
Missing	0 (0)	0 (0)		22 (4)	
Marital status^‖^, *n* (%)			0.303^§^		n.a.
Relationship	24 (62)	22 (73)		–	
No relationship	15 (38)	8 (27)		–	
Marital status^‖^, *n* (%)			n.a.		n.a.
Not married and not living together	–	–		299 (59)	
Married or living together	–	–		192 (38)	
Missing	–	–		17 (3)	

^†^Mann–Whitney *U* test; ^‡^Fisher’s exact test; ^§^Chi square test; ^‖^answer options regarding marital status of two different questionnaires were non-mergeable, therefore these are shown separately. Missing values were excluded for statistical testing (pairwise deletion). Continuous variables are presented as median (range). *Indicates a statistically significant difference (*P* < 0.01).

CCS, childhood cancer survivors; DTC, differentiated thyroid carcinoma; n.a., not applicable.

All DTC survivors and peer controls had the Dutch nationality. Ninety-seven percent of the comparison group had the Dutch nationality; this was not significantly different from the DTC survivors (*P* = 0.558).

#### Psychosocial development

Because scores on psychosocial development did not differ between males and females in all evaluated groups (data not shown), no correction for sex was performed.

*Scale scores*: Scores of the survivors of childhood DTC on all three developmental milestone scales (i.e. social development, autonomy development and psychosexual development) did not differ significantly from those of peer controls or of the comparison group (*P* = 0.592, *P* = 0.084, *P* = 0.841, *P* = 0.233, *P* = 0.241, and *P* = 0.556, respectively, Table 2).

**Table 2:** Scores on psychosocial developmental domains: comparison of survivors of childhood DTC with individuals non-affected with cancer.

	DTC survivors	Peer controls	*P* value	Comparison group	*P* value
*n*	39	30		508	
Social development^†^	22 (20.3, 23)	22 (18, 23)	0.592	21 (19, 23)	0.233
Autonomy development^‡^	9 (8, 10)	10 (9, 11)	0.084	9 (8, 11)	0.241
Psychosexual development^§^	8 (6.5, 8)	8 (6, 8)	0.841	8 (7, 8)	0.556

Scores are shown as median (p25, p27). Comparisons between DTC survivors and other groups were performed using Mann–Whitney *U* tests. Higher scores indicate earlier achievement or achievement of more psychosocial developmental milestones; ^†^scale ranges 12–24; ^‡^scale ranges 6–12; ^§^scale ranges 4–8. Missing values were excluded from statistical testing (pairwise deletion).

CCS, childhood cancer survivors; DTC, differentiated thyroid carcinoma.

*Item scores*: Item scores regarding social development, autonomy development and psychosexual development during or after secondary school did not differ between survivors of childhood DTC and the peer controls or the comparison group (Supplementary Table 1a, b and c).

### II) Comparison to survivors of other types of childhood cancer

#### Sample characteristics

Age at diagnosis was significantly different between survivors of childhood DTC and other CCS (15.6 vs 6.3 years old, respectively, *P* < 0.001); the large majority (35 out of 39, 90%) of survivors of childhood DTC were diagnosed during secondary school. We therefore chose to focus primarily on survivors (DTC and other CCS) diagnosed at age ≥12 years (the age at which Dutch children generally start secondary school).

Thirty-five of the DTC survivors were diagnosed at age ≥12 years (Supplementary Fig. 1). Of the 350 CCS non-affected with thyroid cancer 76 were diagnosed at age ≥12 years.

As shown in Table 3, the median age of these 35 DTC survivors at diagnosis was 15.8 years (range 12.0–18.7), and the median age at diagnosis of CCS was 14.0 years (range 12.0–17.0, *P* < 0.001). The group of DTC survivors included significantly more females than the CCS (91% vs 55%, *P* < 0.001). Median follow-up period for DTC survivors was 10.7 years (range 5.0–23.3), and for the CCS, it was 12.0 years (range 6.2–18.1, *P* = 0.088). DTC survivors and CCS did not differ significantly on other characteristics. Ninety-seven percent of the CCS group were Dutch; this was not significantly different from the DTC survivors (*P* = 1.000).

**Table 3:** Characteristics of survivors of childhood DTC vs survivors of other childhood cancers (both diagnosed age ≥12 years old).

	DTC survivors	Other CCS	*P* value
*n*	35	76	
At diagnosis			
Age (years)	15.8 (12.0–18.7)	14.0 (12.0–17.0)	<0.001*^†^
Sex, *n* (%)			<0.001*^§^
Female	32 (91)	42 (55)	
Male	3 (9)	34 (45)	
At follow-up			
Age at evaluation (years)	26.3 (18.8–35.7)	26.2 (18.9–31.1)	0.741^†^
Follow-up period (years)	10.7 (5.0–23.3)	12.0 (6.2–18.1)	0.088^†^
Employment, *n* (%)			1.000^‡^
Employed and/or student	34 (97)	72 (95)	
Not employed and no student	1 (3)	3 (4)	
Missing	0 (0)	1 (1)	
Completed education, *n* (%)			0.237^§^
Low level	6 (17)	15 (20)	
Medium level	13 (37)	36 (47)	
High level	16 (46)	21 (28)	
Missing	0 (0)	4 (5)	
Marital status^‖^, *n* (%)			n.a.
Relationship	23 (66)	–	
No relationship	12 (34)	–	
Not married and not living together	–	31 (55)	
Married or living together	–	31 (41)	
Missing	–	3 (4)	

^†^Mann–Whitney *U* test; ^‡^Fisher’s exact test; ^§^Chi square test; ^‖^answer options regarding marital status of two different questionnaires were non-mergeable, therefore these are shown separately. Missing values were excluded for statistical testing (pairwise deletion). Continuous variables are presented as median (range). *Indicates a statistically significant difference (*P* < 0.01).

CCS, childhood cancer survivors; DTC, differentiated thyroid carcinoma; n.a., not applicable.

#### Psychosocial development

*Scale scores*: DTC survivors scored higher on social development than did CCS (both diagnosed at ≥12 years, 22 (21, 23) vs 21 (19, 22) out of 24, respectively, *P* = 0.005). DTC survivors also scored higher on social development compared to survivors of childhood leukemia diagnosed at age ≥12 years (22 (21, 23) vs 20 (18, 22) respectively, *P* = 0.001), but their scores were not significantly different from those of survivors of solid tumors or brain tumors. Scale scores of CCS and subgroups of CCS, compared to DTC survivors, did not differ on autonomy development and psychosexual development ( Table 4).

**Table 4:** Scores on psychosocial developmental domains: comparison of survivors of childhood DTC with survivors of other childhood cancers (both diagnosed age ≥12 years old).

	DTC survivors	Childhood cancer survivors (CCS)							
		Total CCS	*P* value	Leukemia/lymphoma	*P* value	Solid tumors	*P* value	Brain tumors	*P* value
*n*	35	76		45		23		8	
Social development^†^	22 (21, 23)	21 (19, 22)	**0.005**	20 (18, 22)	**0.001**	22 (19, 23)	0.164	21.5 (20.3, 22.8)	0.391
Autonomy development^‡^	9 (8, 10)	9 (8, 10)	0.688	10 (8, 10)	0.753	9 (8, 10.5)	0.677	9 (7.5, 11)	0.890
Psychosexual development^§^	8 (7, 8)	7 (6, 8)	0.020	7 (6, 8)	0.040	7 (6, 8)	0.083	5.5 (4.3, 8)	0.097

Scores are shown as median (p25, p27). Comparisons between DTC survivors and other groups were performed using Mann–Whitney *U* tests. Higher scores indicate earlier achievement or achievement of more psychosocial developmental milestones; ^†^scale ranges 12–24; ^‡^scale ranges 6–12; ^§^scale ranges 4–8. Missing values were excluded from statistical testing (pairwise deletion). *P* values in bold indicate a significant value (*P* < 0.01).

CCS, childhood cancer survivors; DTC, differentiated thyroid carcinoma.

**Figure 1:**
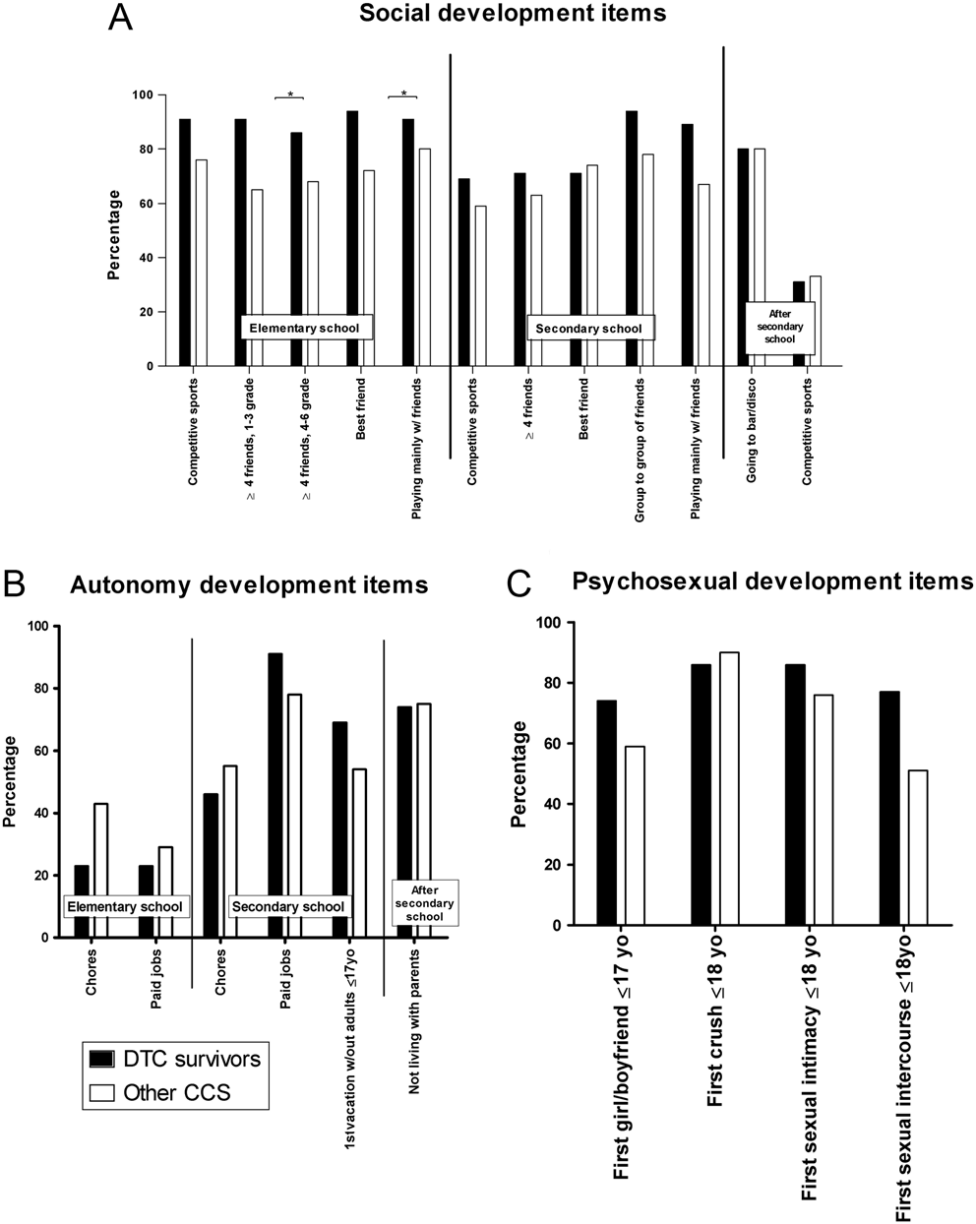
Scores of survivors of childhood DTC (*n* = 35) and childhood cancer survivors (CCS, *n* = 76) of other types of cancer, both diagnosed at age ≥12 years old, on individual items of the CoLQ. Scores indicate the percentage of the group that has reached the developmental milestone. Dark bars: DTC survivors, light bars: other CCS. An asterisk (*) indicates a *P* value <0.01.

*Item scores*: Given the age at diagnosis of DTC survivors, only items that apply to the period during or after secondary school are discussed. Item scores regarding social development, autonomy development, and psychosexual development during or after secondary school did not differ between survivors of DTC and CCS diagnosed at ≥12 years old (Fig. 1 and Supplementary Table 2a, b and c).

Characteristics and psychosocial development scale scores of DTC survivors and CCS diagnosed at all ages are shown in Supplementary Tables 3 and 4.

### Possible determinants of psychosocial development

Disease- treatment and, follow-up characteristics were not significantly associated with scores on domains of psychosocial development (Supplementary Table 5).

## Discussion

The current study shows similar achievement of psychosocial developmental milestones in long-term survivors of childhood DTC as compared to non-affected groups. A slightly better social development in DTC survivors was observed compared to other CCS; differences with leukemia survivors were the most pronounced. Medical characteristics were not associated with a better or worse psychosocial development. Altogether, this indicates that even though the diagnosis of DTC during childhood is a life-altering event, the disease does not seem to have consequences related to altered psychosocial development. Current results align with the overall normal QoL reported in a previous study describing the same cohort (19).

Current results imply that survivors of childhood DTC may be less restricted in their psychosocial development than other CCS; previous studies report hampered psychosocial development in other CCS compared to a non-affected comparison group of similar sex and age (7). This indicates that the degree to which a child is hindered in his or her development depends on the type of cancer (13). For instance, survivors of brain tumors and CCS treated with neurotoxic treatment (i.e. cranial irradiation, intrathecal chemotherapy and specific intravenous chemotherapies) were found to be most vulnerable to impairment of psychosexual and social development as compared to other CCS (10, 11, 13, 20). However, this finding was not confirmed in the current study. This could be due to the fact that results in the current study were based on brain tumor survivors diagnosed during secondary school: brain tumor survivors diagnosed at younger ages were not represented while younger age at diagnosis of a brain tumor is associated with more developmental impairment (21).

Current results allow for conclusions regarding the majority of survivors of childhood DTC that was diagnosed during secondary school. Diagnosis of childhood DTC before secondary school occurs less frequently, therefore, concise conclusions cannot be made.

Factors that could interfere with psychosocial development (e.g. sex, age at diagnosis, age at follow-up, or follow-up duration Supplementary Table 5) were evaluated, but surprisingly showed no significant associations with psychosocial development. It may be that other, not investigated, factors may have played a role.

A first possible explanation for the normal psychosocial development of survivors of childhood DTC could lie in the excellent prognosis of the disease. In addition, the indolent course of DTC allows for flexibility in treatment, thus accommodating school schedules. In general, attending school benefits social development. The more favorable social development in DTC survivors compared to other CCS emphasizes this explanation. However, this statement remains speculative since we did not study school attendance. DTC treatment modalities differ from those of other types of cancer, using chemotherapeutics administered over longer periods of time. Another explanation could be the age upon diagnosis of most children with DTC. Relatively older upon diagnosis, these children as well as their parents have already experienced considerable developmental progression. For example, the foundations for social interactions (friendly as well as romantic) have already been formed. Lastly, fluctuations in thyroid hormone levels during periods of thyroid hormone withdrawal or long-term treatment effects of DTC, such as a weakened voice or the need for medication monitoring, may have less impact on psychosocial development than the aforementioned neurotoxicity and other physical or mental sequelae involved in other childhood cancer treatments (9, 10, 12, 22). It is common practice to substitute DTC survivors with levothyroxine after initial treatment and only use triiodothyronine in preparation for treatment with 131-I (23). As a result, current results suggest that neurological development of childhood DTC survivors is not affected by this approach to treatment.

### Strengths and limitations

There is a great lack of knowledge regarding the long-term impact of childhood thyroid cancer on psychosocial domains (23). A strength of the current study is that it is the first to evaluate psychosocial development in a, though relatively small, unique cohort of survivors of childhood DTC. Using various groups for comparison allowed psychosocial development in these survivors to be placed in different perspectives. However, when interpreting the results, one must keep in mind the limitations of the study. One cannot use the results of this cross-sectional design in long-term survivors to elaborate about psychosocial development in the first 5 years after diagnosis, but eventual long-term results are promising. Not all predictors of psychosocial development were evaluated; for instance, we did not include the dependency of autonomy development on parenting behavior. Moreover, since most DTC survivors were diagnosed during secondary school, the items regarding elementary school were less relevant for the current population. However, the CoLQ is only validated containing all items (7).

### Clinical implications

Current results may be reassuring for children newly diagnosed with DTC, and for their families and caregivers, regarding the possible psychosocial impact of their disease. However, the results do not imply that physicians need not monitor problems with psychosocial development in these survivors. This study presents data of a group of survivors, but individual differences should not be overlooked. Presenting patients with thyroid cancer as a *good cancer* makes them feel that physicians are downplaying their cancer experiences (24).

In conclusion, the current study aimed to evaluate the achievement of psychosocial developmental milestones in survivors of childhood DTC and found no delay in autonomy, social, and psychosexual domains after diagnosis compared to individuals non-affected with cancer. It did find a slightly more favorable development in DTC survivors compared to other CCS. However, before drawing definite conclusions, current findings need to be confirmed in subsequent studies.

## Supplementary Material

Supporting Table 1

Supporting Table 2

Supporting Table 3

Supporting Table 4

Supporting Table 5

Supporting Table 6

Supporting Table 7

Supporting Table 8

Supporting Table 9

## Declaration of interest

The authors declare that there is no conflict of interest that could be perceived as prejudicing the impartiality of the research reported.

## Funding

This work was supported by Stichting Kinderen Kankervrij (the Netherlands, Foundation Children Cancer-Free, Project 81). C M R is supported by the Dutch Cancer Society.

## Author contribution statement

Marloes Nies: conceptualization, methodology, validation, software, formal analysis, investigation, data curation resources, writing – original draft, writing – review and editing, visualization. Bernadette L Dekker: methodology, validation, resources, writing – original draft, writing – review and editing. Esther Sulkers: conceptualization, resources, writing – original draft, writing – review and editing, supervision. Gea A Huizinga: conceptualization, methodology, validation, resources, writing – original draft, writing – review and editing, supervision. Mariëlle S Klein Hesselink: conceptualization, methodology, validation, investigation, data curation, writing – review and editing. Heleen Maurice-Stam: software, investigation, resources, writing – review and editing. Martha A Grootenhuis: investigation, resources, writing – review and editing. Adrienne H Brouwers: writing – review and editing. Johannes G M Burgerhof: software, formal analysis, writing – review and editing. Eveline W C M van Dam: resources, writing – review and editing. Bas Havekes: resources, writing – review and editing. Marry M van den Heuvel-Eibrink: resources, writing – review and editing. Eleonora P M Corssmit: resources, writing – review and editing. Leontien C M Kremer: writing – review and editing. Romana T Netea-Maier: resources, writing – review and editing. Heleen J H van der Pal: resources, writing – review and editing. Robin P Peeters: resources, writing – review and editing. John T M Plukker: writing – review and editing. Cécile M Ronckers: conceptualization, writing – review and editing. Hanneke M van Santen: conceptualization, resources, writing – review and editing. Anouk N A van der Horst-Schrivers: resources writing – original draft, writing – review and editing, supervision. Wim J E Tissing: conceptualization, methodology, validation, resources writing – original draft, writing – review and editing, supervision, project administration, funding acquisition. Gianni Bocca: conceptualization, methodology, validation, resources writing – original draft, writing – review and editing, supervision, project administration, funding acquisition. Thera P Links: conceptualization, methodology, validation, resources, data curation, writing – original draft, writing – review and editing, supervision, project administration, funding acquisition.
