# Supplementary material for: Psychosocial development in survivors of childhood differentiated thyroid carcinoma: a cross-sectional study
Source: Eur J Endocrinol. 2017 Dec 18;178(3):215–23. doi: 10.1530/EJE-17-0741 (PMC5811933; doi:10.1530/EJE-17-0741)
Supplement: Supporting Table 1 [file eje-178-215-t001.pdf]

Supplemental Table 1a. Social development in survivors of childhood DTC versus peer controls and comparison group on item level					
	**DTC Survivors** n = 39	**Peer controls** n = 30		**Comparison group** n = 508	
			P value		*P* Value
**At least one year competitive sports, elementary school, n (%)**			.440[1]		0.175[2]
Yes	36 (92)	24 (80)		427 (84)	
No	3 (8)	4 (13)		80 (16)	
Missing	0 (0)	2 (7)		1 (0)	
**Number of friends in kindergarten through third grade, elementary school, n (%)**			.034[2]		**0.007[2]**
Less than 4	6 (15)	11 (37)		187 (37)	
4 or more	33 (85)	18 (60)		319 (63)	
Missing	0 (0)	1 (3)		2 (0)	
**Number of friends in fourth-sixth grade, elementary school, n (%)**			0.534[2]		0.203[2]
Less than 4	8 (21)	8 (27)		156 (31)	
4 or more	30 (77)	21 (70)		349 (69)	
Missing	1 (3)	1 (3)		3 (1)	
**Best friend, elementary school, n (%)**			0.156[1]		0.011[2]
Yes	36 (92)	23 (77)		377 (74)	
No	3 (8)	6 (20)		131 (26)	
Missing	0 (0)	1 (3)		0 (0)	
**Most of time playing with … elementary school, n (%)**			0.066[2]		1.000[1]
Friends	34 (87)	20 (67)		436 (86)	
Brothers and/or sisters, parents, on your own	5 (13)	9 (30)		62 (12)	
Missing	0 (0)	1 (3)		10 (2)	
**At least one year competitive sports, middle and/or high school, n (%)**			0.800[2]		0.809[2]
Yes	28 (72)	20 (67)		373 (73)	
No	11 (28)	9 (30)		134 (26)	
Missing	0 (0)	1 (3)		1 (0)	
**Number of friends, middle and/or high school, n (%)**			0.547[2]		0.965[2]
Less than 4	12 (31)	7 (23)		154 (30)	
4 or more	27 (69)	22 (73)		352 (69)	
Missing	0 (0)	1 (3)		2 (0)	
**Best friend, middle and/or high school, n (%)**			0.853[2]		0.740[2]
Yes	27 (69)	20 (67)		372 (73)	
No	11 (28)	9 (30)		134 (26)	
Missing	1 (3)	1 (3)		2 (0)	
**Belonging to a group of friends, middle and/or high school, n (%)**			0.574[1]		0.010[2]
Yes	37 (95)	27 (90)		403 (79)	
No	1 (3)	2 (7)		97 (19)	
Missing	1 (3)	1 (3)		8 (2)	
**Leisure time, mainly with … middle and/or high school, n (%)**			0.747[1]		0.602[2]
Friends	32 (82)	25 (83)		430 (85)	
Brothers and/or sisters, parents, on your own	7 (18)	4 (13)		75 (15)	
Missing	0 (0)	1 (3)		3 (1)	
**Going to a bar or disco, middle and/or high school, n (%)**			0.310[2]		0.166[2]
Sometimes / often	29 (74)	25 (83)		430 (85)	
Never	9 (23)	4 (13)		77 (15)	
Missing	1 (3)	1 (3)		1 (0)	
**At least one year competitive sports, after high school, n (%)**			0.197[2]		0.081[2]
Yes	13 (33)	14 (47)		243 (48)	
No	25 (64)	14 (47)		254 (50)	
Missing	1 (3)	2 (7)		11 (2)	

[1] Fisher's Exact test [2] Chi squares test. *P* Values in bold are *P* values <0.01
