# Supplementary material for: Psychosocial development in survivors of childhood differentiated thyroid carcinoma: a cross-sectional study
Source: Eur J Endocrinol. 2017 Dec 18;178(3):215–23. doi: 10.1530/EJE-17-0741 (PMC5811933; doi:10.1530/EJE-17-0741)
Supplement: Supporting Table 2 [file eje-178-215-t002.pdf]

Supplemental Table 1b. Autonomy development in survivors of childhood DTC versus peer controls and comparison group on item level

	DTC Survivors n = 39	Peer controls n = 30	*P* value	Comparison group n = 508	*P* Value
**Regular chores/tasks in your family, elementary school, n (%)**			0.170[2]		0.013[2]
Yes	10 (26)	12 (40)		233 (46)	
No	29 (74)	17 (57)		273 (54)	
Missing	0 (0)	1 (3)		2 (0)	
**Paid jobs, elementary school, n (%)**			0.278[2]		0.309[2]
Yes	10 (26)	11 (37)		170 (33)	
No	29 (74)	18 (60)		336 (66)	
Missing	0 (0)	1 (3)		2 (0)	
**Regular chores/tasks in your family, middle and/or high school, n (%)**			0.038[2]		0.042[2]
Yes	17 (44)	20 (67)		304 (60)	
No	22 (56)	9 (30)		201 (40)	
Missing	0 (0)	1 (3)		3 (1)	
**Paid jobs, middle and/or high school, n (%)**			0.715[1]		0.805[1]
At the age of 18 or younger	35 (90)	25 (83)		443 (87)	
At the age of 19 or older / never	4 (10)	4 (13)		64 (13)	
Missing	0 (0)	1 (3)		1 (0)	
**First time vacation without adults, n (%)**			0.213[2]		0.096[2]
At the age of 17 or younger	26 (67)	15 (50)		268 (53)	
At the age of 18 or older / never	13 (33)	14 (47)		239 (47)	
Missing	0 (0)	1 (3)		1 (0)	
**Leaving parents' home, n (%)**			0.156[2]		0.362[2]
Not living with parents	28 (72)	25 (83)		328 (64)	
Still living with parents	11 (28)	4 (13)		180 (35)	
Missing	0 (0)	1 (3)		0 (0)	

¹ Fisher's Exact test ² Chi squares test. *P* Values in bold are *P* values <0.01
