# Supplementary material for: Psychosocial development in survivors of childhood differentiated thyroid carcinoma: a cross-sectional study
Source: Eur J Endocrinol. 2017 Dec 18;178(3):215–23. doi: 10.1530/EJE-17-0741 (PMC5811933; doi:10.1530/EJE-17-0741)
Supplement: Supporting Table 3 [file eje-178-215-t003.pdf]

Supplemental Table 1c. Psychosexual development in survivors of childhood DTC versus peer controls and comparison group on item level

	**DTC Survivors** n = 39	**Peer controls** n = 30	*P* value	**Comparison group** n = 508	*P* Value
**First girlfriend / boyfriend, n (%)**			0.907[2]		0.316[2]
At the age of 17 or younger	28 (72)	21 (70)		407 (80)	
At the age of 18 or older / never	10 (26)	8 (27)		99 (19)	
Missing	1 (3)	1 (3)		2 (0)	
**First time falling in love, n (%)**			0.224[1]		0.362[1]
At the age of 18 or younger	33 (85)	28 (93)		462 (91)	
At the age of 19 or older / never	5 (13)	1 (3)		42 (8)	
Missing	1 (3)	1 (3)		4 (1)	
**First time sexual intimacy, n (%)**			0.986[2]		0.534[2]
At the age of 18 or younger	31 (80)	23 (77)		421 (83)	
At the age of 19 or older / never	8 (21)	6 (20)		84 (17)	
Missing	0 (0)	1 (3)		4 (1)	
**First time sexual intercourse, n (%)**			0.746[2]		0.189[2]
At the age of 18 or younger	27 (69)	19 (63)		296 (58)	
At the age of 19 or older / never	12 (31)	10 (33)		210 (41)	
Missing	0 (0)	1 (3)		2 (0)	

¹ Fisher's Exact test ² Chi squares test. *P* Values in bold are *P* values <0.01
