# Supplementary material for: Psychosocial development in survivors of childhood differentiated thyroid carcinoma: a cross-sectional study
Source: Eur J Endocrinol. 2017 Dec 18;178(3):215–23. doi: 10.1530/EJE-17-0741 (PMC5811933; doi:10.1530/EJE-17-0741)
Supplement: Supporting Table 4 [file eje-178-215-t004.pdf]

Supplemental Table 2a. Social development in survivors of childhood DTC versus other childhood cancer survivors (diagnosed at age ≥12 years) on item level	DTC Survivors n = 35	Childhood Cancer Survivors n = 76	*P* Value
**At least one year competitive sports, elementary school, n (%)**			0.059[2]
Yes	32 (91)	58 (76)	
No	3 (9)	18 (24)	
Missing	0 (0)	0 (0)	
**Number of friends in kindergarten through third grade, elementary school, n (%)**			**0.005**[2]
Less than 4	3 (9)	25 (33)	
4 or more	32 (91)	49 (65)	
Missing	0 (0)	2 (3)	
**Number of friends in fourth-sixth grade, elementary school, n (%)**			0.054[2]
Less than 4	5 (14)	24 (32)	
4 or more	30 (86)	52 (68)	
Missing	0 (0)	0 (0)	
**Best friend, elementary school, n (%)**			**0.008**[2]
Yes	33 (94)	55 (72)	
No	2 (6)	21 (28)	
Missing	0 (0)	0 (0)	
**Most of time playing with ….., elementary school, n (%)**			0.334[1]
Friends	32 (91)	61 (80)	
Brothers and/or sisters, parents, on your own	3 (9)	11 (14)	
Missing	0 (0)	4 (5)	
**At least one year competitive sports, middle and/or high school, n (%)**			0.345[2]
Yes	24 (69)	45 (59)	
No	11 (31)	31 (41)	
Missing	0 (0)	0 (0)	
**Number of friends, middle and/or high school, n (%)**			0.394[2]
Less than 4	10 (29)	28 (37)	
4 or more	25 (71)	48 (63)	
Missing	0 (0)	0 (0)	
**Best friend, middle and/or high school, n (%)**			0.986[2]
Yes	25 (71)	56 (74)	
No	9 (26)	20 (26)	
Missing	1 (3)	0 (0)	
**Belonging to a group of friends, middle and/or high school, n (%)**			0.011[2]
Yes	33 (94)	59 (78)	
No	1 (3)	17 (22)	
Missing	1(3)	0 (0)	
**Leisure time, mainly with ….., middle and/or high school, n (%)**			0.026[2]
Friends	31 (89)	51 (67)	
Brothers and/or sisters, parents, on your own	4 (11)	23 (30)	
Missing	0 (0)	2 (3)	
**Going to a bar or disco, middle and/or high school, n (%)**			0.797[2]
Sometimes / often	28 (80)	61 (80)	
Never	6 (17)	15 (20)	
Missing	1 (3)	0 (0)	
**At least one year competitive sports, after high school, n (%)**			0.920[2]
Yes	11 (31)	25 (33)	
No	23 (66)	50 (66)	
Missing	1 (3)	1 (3)	

¹ Fisher's Exact test ² Chi squares test. *P* Values in bold are *P* values <0.01
