# Supplementary material for: Psychosocial development in survivors of childhood differentiated thyroid carcinoma: a cross-sectional study
Source: Eur J Endocrinol. 2017 Dec 18;178(3):215–23. doi: 10.1530/EJE-17-0741 (PMC5811933; doi:10.1530/EJE-17-0741)
Supplement: Supporting Table 5 [file eje-178-215-t005.pdf]

**Supplemental Table 2b. Autonomy development in survivors of childhood DTC versus other childhood cancer survivors (diagnosed at age ≥12 years) on item level**

| | DTC Survivors n = 35 | Childhood Cancer Survivors n = 76 | *P* Value |
|---|---|---|---|
| **Regular chores/tasks in your family, elementary school, n (%)** | | | 0.033[2] |
| Yes | 8 (23) | 33 (43) | |
| No | 27 (77) | 42 (55) | |
| Missing | 0 (0) | 1 (1) | |
| **Paid jobs, elementary school, n (%)** | | | 0.477[2] |
| Yes | 8 (23) | 22 (29) | |
| No | 27 (77) | 53 (70) | |
| Missing | 0 (0) | 1 (1) | |
| **Regular chores/tasks in your family, middle and/or high school, n (%)** | | | 0.349[2] |
| Yes | 16 (46) | 42 (55) | |
| No | 19 (54) | 34 (45) | |
| Missing | 0 (0) | 0 (0) | |
| **Paid jobs, middle and/or high school, n (%)** | | | 0.097[2] |
| At the age of 18 or younger | 32 (91) | 59 (78) | |
| At the age of 19 or older / never | 3 (9) | 17 (22) | |
| Missing | 0 (0) | 0 (0) | |
| **First time vacation without adults, n (%)** | | | 0.146[2] |
| At the age of 17 or younger | 24 (69) | 41 (54) | |
| At the age of 18 or older / never | 11 (31) | 35 (46) | |
| Missing | 0 (0) | 0 (0) | |
| **Leaving parents' home, n (%)** | | | 0.936[2] |
| Not living with parents | 26 (74) | 57 (75) | |
| Still living with parents | 9 (26) | 19 (25) | |
| Missing | 0 (0) | 0 (0) | |

¹ Fisher's Exact test ² Chi squares test. *P* Values in bold are *P* values <0.01
