# Supplementary material for: Psychosocial development in survivors of childhood differentiated thyroid carcinoma: a cross-sectional study
Source: Eur J Endocrinol. 2017 Dec 18;178(3):215–23. doi: 10.1530/EJE-17-0741 (PMC5811933; doi:10.1530/EJE-17-0741)
Supplement: Supporting Table 6 [file eje-178-215-t006.pdf]

Supplemental Table 2c. Psychosexual development in survivors of childhood DTC versus other childhood cancer survivors (diagnosed at age ≥12 years) on item level

	DTC Survivors n = 35	Childhood Cancer Survivors n = 76	*P* Value
**First girlfriend / boyfriend, n (%)**			0.080[2]
At the age of 17 or younger	26 (74)	45 (59)	
At the age of 18 or older / never	8 (23)	31 (41)	
Missing	1 (3)	0 (0)	
**First time falling in love, n (%)**			0.696[1]
At the age of 18 or younger	30 (86)	68 (90)	
At the age of 19 or older / never	4 (11)	7 (9)	
Missing	1 (3)	1 (1)	
**First time sexual intimacy, n (%)**			0.256[2]
At the age of 18 or younger	30 (86)	58 (76)	
At the age of 19 or older / never	5 (14)	18 (24)	
Missing	0 (0)	0 (0)	
**First time sexual intercourse, n (%)**			0.010[2]
At the age of 18 or younger	27 (77)	39 (51)	
At the age of 19 or older / never	8 (23)	37 (49)	
Missing	0 (0)	0 (0)	

¹ Fisher's Exact test ² Chi squares test. *P* Values in bold are *P* values <0.01
