# Supplementary material for: Psychosocial development in survivors of childhood differentiated thyroid carcinoma: a cross-sectional study
Source: Eur J Endocrinol. 2017 Dec 18;178(3):215–23. doi: 10.1530/EJE-17-0741 (PMC5811933; doi:10.1530/EJE-17-0741)
Supplement: Supporting Table 7 [file eje-178-215-t007.pdf]

Supplemental Table 3. Characteristics of survivors of childhood DTC versus survivors of other childhood cancers (diagnosed at all ages)

	**DTC Survivors** n = 39	**Childhood Cancer Survivors** n = 350[ǁ]	*P* Value
**At diagnosis**			
**Age at diagnosis, y**	15.6 (8.7-18.7)	6.3 (0.0-17.0)	**0.001**[§]
**Sex, n (%)**			**<0.001**[‡]
Female	34 (87)	176 (50)	
Male	5 (13)	175 (50)	
**At follow-up**			
**Age at evaluation, y**	26.2 (18.8-35.7)	24.4 (17.7-31.1)	0.021[§]
**Follow-up period, y**	10.7 (5.0-25.3)	16.9 (6.2-30.7)	**<0.001**[§]
**Employment, n (%)**			0.338[†]
Employed and/or student	38 (97)	318 (91)	
Not employed and no student	1 (3)	27 (8)	
Missing	0 (0)	5 (1)	
**Completed education, n (%)**			**<0.001**[‡]
Low level	7 (18)	113 (32)	
Medium level	15 (39)	167 (48)	
High level	17 (44)	54 (15)	
Missing	0 (0)	16 (5)	
**Marital status[¶], n (%)**			n.a.
Relationship	24 (62)	-	
No relationship	15 (38)	-	
**Marital status[¶], n (%)**			n.a.
Not married and not living together	-	234 (67)	
Married or living together	-	104 (30)	
Missing	-	12 (3)	

† Fisher's Exact Test ‡ Chi square test § Mann Whitney U test. ǁ n = 350, three childhood cancer survivors diagnosed with thyroid cancer were removed for analyses (two had thyroid carcinoma as primary malignancy, one had thyroid cancer as second malignant neoplasm); ¶' answer options regarding marital status of two different questionnaires were non-mergeable, therefore these are shown separately. Missing values were excluded for statistical testing (pairwise deletion). Continuous variables are presented as median (range). *P* Values in bold indicate a significant difference (*P* < 0.01). Abbreviations: DTC, differentiated thyroid carcinoma; n.a., not applicable.
