# Supplementary material for: Psychosocial development in survivors of childhood differentiated thyroid carcinoma: a cross-sectional study
Source: Eur J Endocrinol. 2017 Dec 18;178(3):215–23. doi: 10.1530/EJE-17-0741 (PMC5811933; doi:10.1530/EJE-17-0741)
Supplement: Supporting Table 8 [file eje-178-215-t008.pdf]

Supplemental Table 4. Scores on psychosocial developmental domains of survivors of childhood DTC versus survivors of other childhood cancers (diagnosed at all ages)

	DTC survivors	Childhood Cancer Survivors (CCS)							
		**Total CCS**		**Leukemia/ lymphoma**		**Solid tumors**		**Brain tumors**	
	n = 39	n = 350		n = 175		n = 150		n = 25	
			P Value		*P* Value		*P* Value		*P* Value
**Social development** †	22 (20.3, 23)	21 (18, 22)	**0.008**	20 (18, 22)	**0.004**	21 (19, 23)	0.068	20 (17, 22)	**0.003**
**Autonomy development** ‡	9 (8, 10)	9 (8, 10)	0.734	9 (8, 10)	0.693	9 (8, 10)	0.974	9 (8, 10)	0.278
**Psychosexual development** §	8 (6.5, 8)	7 (5, 8)	0.036	7 (5, 8)	0.042	7 (6, 8)	0.097	5 (4, 8)	**0.007**

Scores are shown as median (p25, p27). Comparisons between DTC survivors and other groups were performed using Mann Whitney U tests. Higher score indicates achievement of earlier or more psychosocial developmental milestones; †, scale ranges 12-24; ‡, scale ranges 6-12; §, scale ranges 4-8. Missing values were excluded for statistical testing. *P* values in bold indicate a significant value (*P* <0.01). Abbreviation: DTC, differentiated thyroid carcinoma; CCS, childhood cancer survivors.
