# Supplementary material for: Psychosocial development in survivors of childhood differentiated thyroid carcinoma: a cross-sectional study
Source: Eur J Endocrinol. 2017 Dec 18;178(3):215–23. doi: 10.1530/EJE-17-0741 (PMC5811933; doi:10.1530/EJE-17-0741)
Supplement: Supporting Table 9 [file eje-178-215-t009.pdf]

Supplemental Table 5. Medical characteristics associated with psychosocial development scales in survivors of childhood DTC

	Social development		Autonomy development		Psychosexual development	
		P Value		*P* Value		*P* Value
**Survivor characteristics**						
**Sex, n (%)**		0.790[†]		0.107[†]		0.949[†]
Females (n = 34)	22		9		8	
Males (n = 5)	22		8		8	
**Age at evaluation, years**		0.946[‡]		0.043[‡]		0.713[‡]
Correlation coefficient	-0.012		0.325		0.063	
**Tumor characteristics**						
**Age at diagnosis, years**		0.691[‡]		0.816[‡]		0.140[‡]
Correlation coefficient	0.069		0.038		0.248	
**T stage, n (%)**		0.676[†]		0.578[†]		0.224[†]
T1-2 (n = 27)	22		9		8	
T3-4 (n = 10)	23		10		6	
**N stage, n (%)**		0.539[†]		0.989[†]		0.772[†]
N0 (n = 16)	23		9		8	
N1 (n = 23)	22		9		8	
**M stage, n (%)**		0.292[†]		0.474[†]		0.350[†]
M0 (n = 34)	22		9		8	
M1 (n = 5)	21		9		6	
**Histology, n (%)**		0.450[†]		0.488[†]		0.293[†]
PTC (n = 35)	22		9		8	
FTC (n = 4)	23		9		8	
**Treatment characteristics**						
**Permanent hypoparathyroidism, n (%)**		0.428[†]		0.832[†]		0.428[†]
Yes (n = 10)	21		10		8	
No (n = 28)	22		10		8	
**RLN injury, n (%)**		0.955[†]		0.450[†]		0.565[†]
Yes (n = 4)	22		9		7	
No (n = 32)	22		9		8	
**Cumulative 131-I dose, MBq**		0.511[‡]		0.114[‡]		0.946[‡]
Correlation coefficient	0.113		0.257		0.012	
**Follow-up characteristics**						
**TSH suppression, mU/l**		0.448[‡]		0.858[‡]		0.396[‡]
Correlation coefficient	0.129		0.035		0.142	
**Follow-up duration, years**		0.615[‡]		0.038[‡]		0.700[‡]
Correlation coefficient	-0.087		0.334		-0.065	
**Outcome, n (%)**		0.043[§]		0.539[§]		0.789[§]
Remission (n = 32)	22		9		8	
Recurrence (n = 3)	23		12		8	
Persistent disease (n = 4)	24		10		8	

Scores are presented as medians, except when otherwise specified. † Mann-Whitney U test ‡ Spearman's rank correlation coefficient § Kruskal-Wallis test. *P* Values in bold are *P* values <0.01. Abbreviations: DTC, differentiated thyroid carcinoma; TNM stage (according to 7th ed. Of American Joint Committee[a]), tumor node metastases stage; RLN, recurrent laryngeal nerve; 131-I, radioactive iodine; MBq, megabecquerel; TSH, thyrotropin; mU/l, milliunits per liter.

[a] Sobin LH, Gospodarowicz MK, Wittekind C, eds. TNM Classification of Malignant Tumours. 7th ed. Oxford, United Kingdom: Wiley-Blackwell; 2009.
